# Cardiovascular Imaging in Obesity

**DOI:** 10.3390/nu13030744

**Published:** 2021-02-26

**Authors:** Sophie I. Mavrogeni, Flora Bacopoulou, George Markousis-Mavrogenis, George Chrousos, Evangelia Charmandari

**Affiliations:** 1 Onassis Cardiac Surgery Center, 17674 Athens, Greece; sophie.mavrogeni@gmail.com (S.I.M.); georgemm32@gmail.com (G.M.-M.); 2 University Research Institute of Maternal and Child Health and Precision Medicine, and UNESCO Chair on Adolescent Health Care, National and Kapodistrian University of Athens Medical School, ‘Aghia Sophia’ Children’s Hospital, 11527 Athens, Greece; bacopouf@hotmail.com (F.B.); chrousos@gmail.com (G.C.); 3 Division of Endocrinology, Metabolism and Diabetes, First Department of Pediatrics, Νational and Kapodistrian University of Athens Medical School, ‘Aghia Sophia’ Children’s Hospital, 11527 Athens, Greece; 4 Division of Endocrinology and Metabolism, Center for Clinical, Experimental Surgery and Translational Research, Biomedical Research Foundation of the Academy of Athens, 11527 Athens, Greece

**Keywords:** cardiovascular magnetic resonance, obesity, echocardiography, nuclear cardiology, coronary artery computed tomography

## Abstract

Obesity represents one of the most challenging public health problems of our century. It accounts for approximately 5% of deaths worldwide, mostly owing to cardiovascular disease and its associated complications. Cardiovascular noninvasive imaging may provide early accurate information about hypertrophy and ischemia/fibrosis in obese subjects. Echocardiography and nuclear cardiology have serious limitations in obese subjects owing to poor acoustic window and attenuation artifacts, respectively. Coronary computed tomography angiography can provide information about obstructive coronary disease; however, the use of radiation is a serious disadvantage. Finally, cardiac magnetic resonance (CMR) holds the promise of an “all in one” examination by combining evaluation of function, wall motion/thickness, stress rest/perfusion, replacement and diffuse fibrosis without radiation. Future studies are required to document the cost/benefit ratio of the CMR in the evaluation of cardiovascular risk in overweight/obese children and adolescents.

## 1. Introduction

Obesity represents one of the most challenging public health problems of our century owing to both its epidemic proportions worldwide and the associated significant morbidity and mortality. Obesity accounts for approximately 5% of deaths worldwide, mainly due to cardiovascular disease (CVD) and its associated complications [1,2,3]. The adipose tissue is an endocrine organ that influences homeostasis, angiogenesis, immunity, and glucose and lipid metabolism [4]. Furthermore, it leads to low-grade inflammation and increased production of proinflammatory cytokines [5] and is associated with insulin resistance, diabetes mellitus type 2, dyslipidemia, hypertension and endothelial dysfunction [6,7,8]. In addition to the increased morbidity and mortality, obesity accounts for a significant increase in public health costs [9].

Obesity in childhood and adolescence has also reached epidemic proportions worldwide. According to the World Health Organization (WHO), 41 million children under the age of 5 years and more than 340 million children and adolescents aged 5–19 years are overweight or obese [1,2,3]. Overweight or obese children and adolescents are more likely to become obese adults, thereby increasing CVD-related morbidity and mortality in adulthood and leading to a shorter life expectancy [1,2,3,6,7,8]. Many CVD abnormalities observed in obese adults are also observed in obese children, leading to significant changes, including hypertrophy [10], wall motion abnormalities [11] and cardiac steatosis [12].

Obesity promotes the development of CVD due to the increased prevalence of atherosclerosis. It has been documented that atherosclerotic vascular lesions have a more rapid evolution in obese patients compared with patients with normal body mass index (BMI); therefore, obesity represents an independent risk factor of coronary artery disease (CAD) [13,14]. Furthermore, obesity is associated with other medical conditions, including hypertension, diabetes mellitus type 2, insulin resistance and sleep apnea, which also contribute to the rapid development of CAD [15]. However, in the case of preexisting CVD, the mortality of patients with obesity is lower than that of patients with normal BMI, an observation known as the “obesity paradox” with the exact mechanism still not having been clarified. Taking into consideration the increased CVD risk in patients with obesity, it is important to undertake a detailed cardiovascular screening of asymptomatic obese patients in order to ensure early diagnosis and treatment of subclinical CVD [16].

In addition to the above, obesity increases both the aldosterone concentrations and the mineralocorticoid receptor expression, which promote interstitial cardiac fibrosis with concurrent platelet aggregation and endothelial dysfunction, finally leading to heart failure [17,18]. Last, but not least, obesity induces various anatomical/functional changes that play an important role in arrhythmogenesis. These changes include left atrial dilatation/dysfunction, leading to increased incidence of atrial fibrillation [19]. Finally, obesity increases the incidence of sudden cardiac death through cardiac remodeling and QT prolongation [20].

The most typical cardiac change in obese children is the increase in myocardial mass, known as hypertrophy [21]. According to various imaging patterns, cardiac hypertrophy is categorized in concentric and eccentric hypertrophy, characterized by increased mass and wall thickness and increased mass with normal wall thickness, respectively. Of them, concentric hypertrophy is more significantly associated with mortality compared to eccentric hypertrophy and concentric remodeling, which is characterized by normal mass and increased wall thickness [22].

In a study of obese children, 42% of those with normal blood pressure had concentric remodeling and 23% concentric hypertrophy. However, hypertensive, obese children had a two-fold higher incidence of concentric hypertrophy compared to normotensive controls, which is not associated with fractional shortening [23]. It is worth noting that M-mode studies have serious limitations because they are an operator and acoustic window-dependent modalities [24].

Various echocardiographic studies show increase [25], decrease [26], or no changes of left ventricular ejection fraction (LVEF), which is the commonest used parameter in cardiology [27]. As an alternative to LVEF, advanced indices such as strain, torsion, and contraction synchrony are considered to be more sensitive indices of function and future mortality [11]. There is evidence that these parameters may detect systolic alterations early before disease symptoms will be overt, which is ideal for monitoring asymptomatic subjects [28]. Currently, impairment of these indices is found in asymptomatic obese children [29,30].

## 2. Cardiovascular Imaging in Obesity

The noninvasive cardiac imaging modalities used for CVD assessment in obesity are detailed below.

### 2.1. Transthoracic Echocardiography (TTE)

TTE represents the cornerstone of cardiac imaging. It is a widely available, inexpensive, bedside, radiation-free modality with great experience among cardiologists. However, it is limited by the operator and acoustic window dependency [31]. In almost all TTE studies, LVEF was normal or increased in all obesity classes, apart from the cardiac output and workload [32,33]. However, recent studies emphasized the subclinical systolic dysfunction in obese patients with preserved LVEF, using tissue Doppler imaging (TDI), strain rate imaging (SRI), and speckle tracking [34]. Finally, other studies showed that obese patients had lower values of strain rates and torsion with preserved LVEF [35,36,37,38].

It is difficult to evaluate the role of obesity in diastolic function because most of the obese patients are hypertensive, and 30% of them are diabetics. However, it is already known that there is a significant correlation between BMI and left atrial pressure, expressed by the E/Em ratio [35]. Furthermore, obesity is related to mild diastolic dysfunction that increases even more with coexisting cardiovascular risk factors and higher BMI [39,40].

It is important to underline the significance of the left atrium in obesity. Left atrium enlargement usually coexists with LV hypertrophy, and BMI correlates with both left atrial dimensions and progressive dilation during the follow-up, independently of blood pressure [41]. As a result, left atrium enlargement/dysfunction, in association with obesity, is an independent risk factor for atrial fibrillation. Furthermore, obesity, even in the absence of hypertension, leads to reduced atrial deformation that is already present in childhood [42].

Finally, epicardial fat is present between the epicardial and the parietal pericardium, mostly in obese subjects with increased cardiometabolic risk [43]. Several studies have demonstrated that epicardial fat is a better index than the hip/waist ratio to evaluate the carotid stiffness in obese patients [44]. In addition, pericardial fat was associated with a high prevalence of CVD in sex and age-matched controls in a cohort of 1267 subjects [45]. Ideally, epicardial fat can be estimated accurately using computed tomography (CT) and cardiac magnetic resonance (CMR), but it can also be qualitatively assessed by conventional 2D echocardiography. Regarding the imaging of the right ventricle (RV), although its assessment is rather difficult in obese patients, there are TTE data supporting the crescent and irregular shape of the RV. Recently, 3D echocardiography and CMR have overcome the limitations of TTE. In the MESA study enrolling 4127 subjects, CMR showed that obese patients had increased RV mass, end-diastolic volume, stroke volume and decreased RVEF compared with lean subjects, independently of LV dimension and hypertension [46]. Sleep apnea disorders and asymptomatic hypoxic nocturnal episodes may be responsible for the pathogenesis of RV hypertrophy in obese people.

TTE, including the new echocardiographic techniques, can identify early subclinical LV-RV changes related to obesity, but in many cases, the presence of epicardial fat leads to poor acoustic window [47,48]. However, according to the European Association of Cardiovascular Imaging (EACVI) recommendations, a contrast-echocardiographic study should be performed prior to referring an obese patient for a CMR [49]. Using contrast is of great value to assess LV structure/function, as well as segmental wall motion and thickening during stress echocardiography [49].

### 2.2. Nuclear Cardiology (NC)

In nuclear cardiology, if exercise is used as a stress factor, the poor exercise capability of obese subjects may lead to underestimation of stress ischemia. To avoid the limitations of the exercise test, pharmacologic stress factors have been used. In this context, regadenoson, a selective adenosine A_2A_ receptor agonist, has been proposed as the ideal representative of pharmacologic stress. It is a coronary vasodilator agent of immediate and short activities that can be administered as a fixed-dose bolus without modification according to body weight. It is safe for all patient groups and has great efficacy regardless of BMI [50].

Another important issue in nuclear cardiology is the soft tissue attenuation of radioactive tracers leading to artifacts and poor signal-to-noise ratios in single-photon emission computed tomography (SPECT) imaging [51]. Therefore, there is a great need for attenuation correction in order to determine if a perfusion defect is a real finding or artifact. It is also important to clarify if the defect is reversible or is the result of extracardiac tracer activity. Furthermore, gantry bore and table weight may also limit the evaluation of obese people using SPECT or positron emission tomography (PET). The application of PET/CT allows accurate attenuation correction and reduction of false-positive results. Recent findings showed the superiority of flurpiridaz against SPECT for the assessment of CAD in obese subjects using a significantly lower radiation dose [52]. Furthermore, ultrafast, high-efficiency SPECT cameras have improved the SPECT sensitivity showing excellent capability for detection of CAD in highly obese patients [53] by allowing evaluation of patients over 246 kg vs. 180 kg for conventional SPECT. The potential of coronary flow reserve measurement using SPECT may allow the assessment of coronary flow reserve in obese subjects, which cannot be quantified using PET/CT [54].

Obesity can also alter the I-123 metaiodobenzylguanidine indices of cardiac sympathetic innervation in patients with heart failure, supporting a role of obesity in impairing sympathetic innervations [55]. It is evident that not only the assessment of ischemia/fibrosis but also adrenergic alterations can provide important insights about CVD in obese people. However, further studies are required to determine the best nuclear protocol to evaluate CVD in obesity.

### 2.3. Coronary Computed Tomography Angiography (CCTA)

CCTA can provide an accurate, reproducible and noninvasive evaluation of the atherosclerotic, coronary arteries plaques. New imaging algorithms using reduced radiation dose improve the diagnostic capability in heavily calcified arteries of patients with severe obesity or irregular cardiac rhythm. Furthermore, computed tomography myocardial perfusion imaging can quantify myocardial ischemia with simultaneous CCTA acquisition using a low amount of contrast agent and radiation [56]. In severely obese patients, a normal CCTA before bariatric surgery is a good prognostic factor to predict cardiac events in the postoperative period [57]. Recent studies have shown that CCTA using dual-source CT (DSCT) is of high diagnostic accuracy in both obese and normal-BMI subjects [58]. However, the use of nephrotoxic contrast agents and the substantial amounts of ionizing radiation still used in CCTA does not allow its routine application.

### 2.4. The Emerging Role of CMR in Obesity

**Figure 1:**
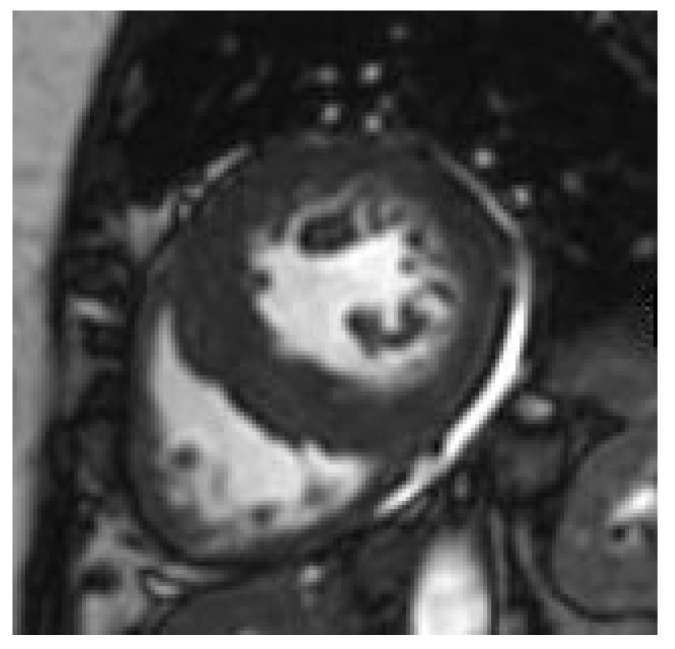
Concentric hypertrophy in an obese adolescent.

**Figure 2:**
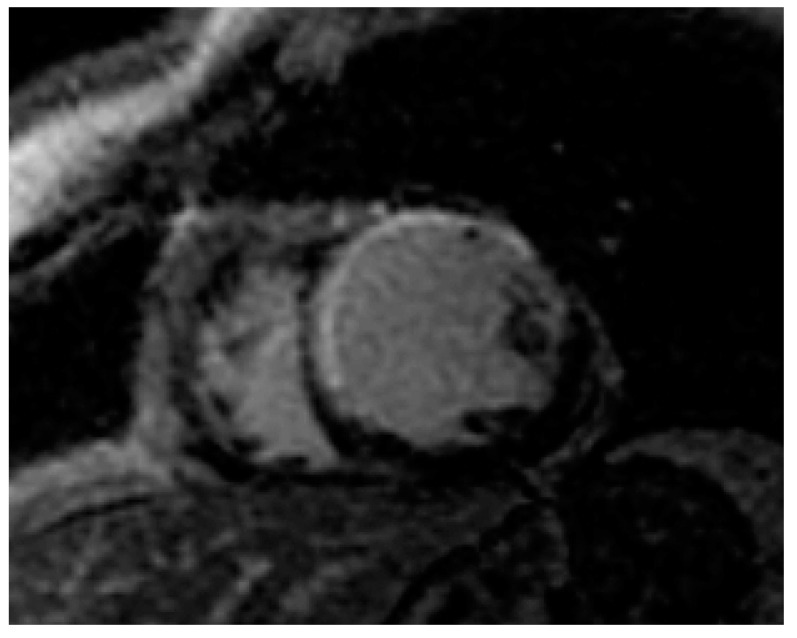
Myocardial infarction (white area) in an obese adult.

The great advantage of CMR is that it can provide direct information about the status of all cardiac tissues noninvasively and without radiation [31]. The basic pulse sequences used by CMR include balanced steady-state free precession (bSSFP) for function and wall motion/thickness evaluation (Figure 1), T2- weighted images (T2-W)/T2 mapping for assessment of edema and T1-weighted images (T1-W) for assessment of morphology. T1 images 15 min after the injection of gadolinium (LGE images) (Figure 2) can reliably identify myocardial fibrosis, which appears as a bright area in nulled, black myocardium “bright is dead”. These images show replacement fibrosis and are similar to pathology images, thus representing the gold standard of noninvasive assessment of fibrosis [31]. However, LGE has inherent disadvantages for the assessment of diffuse fibrosis because its contrast is based on the signal differences between fibrotic and normal myocardium. To overcome this obstacle, a T1 mapping (native or pre-contrast and post-contrast) has been proposed. T1 mapping can detect diffuse myocardial fibrosis missed by the currently used circulating biomarkers [31]. Furthermore, contrast-enhanced T1 mapping is used for the extracellular volume fraction (ECV) calculation together with native T1 mapping.

CMR is the gold standard for quantification of visceral and epicardial fat, which can negatively affect cardiac function [59]. Furthermore, T1-W after pharmacologic stress with adenosine and bolus injection paramagnetic contrast agent can provide accurate, reproducible information about myocardial perfusion during stress [31]. Stress CMR can evaluate ventricular function, stress and rest perfusion/fibrosis in the same examination [60]. Compared to other imaging modalities, stress CMR has high spatial/temporal resolution and is not affected by imaging parameters that influence other imaging modalities. Currently, stress CMR is proven of great value in determining the prognosis of obese patients accurately, with very few patients without ischemia/infarction presenting future events [61]. Furthermore, in a large multicenter registry, obesity did not negatively impact the risk stratification of patients as evaluated by stress CMR [61]. However, lack of availability and high cost still limit its wide use [30]. A comparison between various cardiovascular imaging modalities in obesity is presented in Table 1.

CMR has also been used to evaluate changes in cardiac remodeling, which begins as early as the age of eight years. Obese patients with concentric hypertrophy and impaired strain are at higher risk demanding more intense evaluation [62]. Furthermore, in addition to LV remodeling, overweight and obese children also have RV remodeling. In addition, children with LV concentric hypertrophy also show impaired RV due to interventricular interaction [63].

## 3. Conclusions

Obesity is a significant risk factor for increased morbidity and mortality owing to cardiovascular disease. Cardiovascular noninvasive imaging can provide early accurate information about hypertrophy and ischemia/fibrosis in obese subjects. Echocardiography and nuclear cardiology have serious limitations in patients with obesity owing to poor acoustic window and attenuation artifacts, respectively. CCTA can provide information about obstructive coronary disease; however, the use of radiation is a serious disadvantage. Finally, CMR holds the promise of an “all in one” examination by combining evaluation of function, wall motion/thickness, stress rest/perfusion, replacement and diffuse fibrosis without radiation. Multicenter studies are required to assess the cost/benefit ratio of the routine use of CMR in the early CVD assessment in subjects with obesity.

## Figures and Tables

**Table 1:** Comparison of noninvasive imaging modalities in the evaluation of obese subjects

Modalities	Availability	Cost	Expertise	Radiation	Kidney Toxicity	Acoustic Window Dependency	Operator Dependency	Attenuation Artifacts
**Echo**	++++	+	++++	‒	‒	++++	++++	−
**SPECT/PET**	++++	+++	+++	+++	−	−	−	++++
**CCTA**	+++	+++	+++	+++	+++	−	−	−
**CMR**	++	++++	++	−	++	−	−	−

(+) indicates presence and (−) indicates absence.

## Data Availability

Data sharing not applicable.
